# Associations of Fetal and Infant Weight Change With General, Visceral, and Organ Adiposity at School Age

**DOI:** 10.1001/jamanetworkopen.2019.2843

**Published:** 2019-04-26

**Authors:** Suzanne Vogelezang, Susana Santos, Liza Toemen, Edwin H. G. Oei, Janine F. Felix, Vincent W. V. Jaddoe

**Affiliations:** 1 The Generation R Study Group, Erasmus Medical Center, University Medical Center, Rotterdam, the Netherlands; 2 Department of Paediatrics, Erasmus Medical Center, University Medical Center, Rotterdam, the Netherlands; 3 Department of Epidemiology, Erasmus Medical Center, University Medical Center, Rotterdam, the Netherlands; 4 Department of Radiology and Nuclear Medicine, Erasmus Medical Center, University Medical Center, Rotterdam, the Netherlands

## Abstract

**Importance:**

Both fetal and infant growth influence obesity later in life. The association of longitudinal fetal and infant growth patterns with organ fat is unknown.

**Objective:**

To examine the associations of fetal and infant weight change with general, visceral, and organ adiposity at school age.

**Design, Setting, and Participants:**

This cohort study was embedded in the Generation R Study, a population-based prospective cohort study in Rotterdam, the Netherlands. Pregnant women with a delivery date between April 2002 and January 2006 were eligible to participate. Follow-up measurements were performed for 3205 children. Data analysis of this population was performed from July 26, 2018, to February 7, 2019.

**Exposures:**

Fetal weight was estimated in the second and third trimester of pregnancy. Infant weight was measured at 6, 12, and 24 months. Fetal and infant weight acceleration or deceleration were defined as a change in standard deviation scores greater than 0.67 between 2 ages.

**Main Outcomes and Measures:**

Visceral fat index, pericardial fat index, and liver fat fraction were measured by magnetic resonance imaging.

**Results:**

The sample consisted of 3205 children (1632 girls [50.9%]; mean [SD] age, 9.8 [0.3] years). Children born small for gestational age had the lowest median body mass index compared with children born appropriate for gestational age and large for gestational age (16.4 [90% range, 14.1-23.6] vs 16.9 [90% range, 14.4-22.8] vs 17.4 [90% range, 14.9-22.7]). Compared with children with normal fetal and infant growth (533 of 2370 [22.5%]), those with fetal weight deceleration followed by infant weight acceleration (263 of 2370 [11.1%]) had the highest visceral fat index (standard deviation scores, 0.18; 95% CI, 0.03-0.33; *P* = .02) and liver fat fraction (standard deviation scores, 0.34; 95% CI, 0.20-0.48; *P* < .001).

**Conclusions and Relevance:**

Fetal and infant weight change patterns were both associated with childhood body fat, but weight change patterns in infancy tended to have larger effects. Fetal growth restriction followed by infant growth acceleration was associated with increased visceral and liver fat.

## Key Points

**Question:**

Are fetal and infant weight change associated with general, visceral, and organ fat at school age?

**Findings:**

In this population-based cohort study among 3205 children using magnetic resonance imaging for fat measurements, children with fetal weight deceleration followed by infant weight acceleration showed the highest visceral fat index and liver fat fraction.

**Meaning:**

Different longitudinal fetal and infant weight change patterns may affect visceral body fat and organ fat measures.

## Teaser

This population-based cohort study, a part of the Generation R Study from the Netherlands, examines the associations of fetal and infant weight change with general, visceral, and organ adiposity at school age.

## Introduction

Childhood body fat may be associated with patterns of fetal and infant weight change.^1,2,3^ Children born small for gestational age (SGA) tend to have infant growth acceleration, whereas those born large for gestational age (LGA) tend to have infant growth deceleration.^2,4,5^ A previous study reported that fetal growth deceleration followed by infant growth acceleration may lead to an adverse body fat distribution at age 6 years.^1^ Studies using longitudinal growth data showed that both infant peak weight velocity (PWV), reflecting the greatest infant weight change, and body mass index at adiposity peak (BMIAP), reflecting body mass index (BMI) reached at adiposity peak, may affect childhood adiposity.^6,7,8,9^ Little is known about fetal and infant growth patterns affecting visceral, liver, and pericardial fat, which are strongly associated with cardiometabolic phenotypes.^10,11,12,13^ Identification of early-life growth patterns affecting specific body fat measures from childhood onward may contribute to future prevention strategies.

In a population-based prospective cohort study among 3205 children, we examined the associations of fetal and infant weight change with visceral fat index, pericardial fat index, and liver fat fraction measured by magnetic resonance imaging at age 10 years. We focused specifically on identification of critical periods, combined fetal and infant weight change associations, and associations of PWV, BMIAP, and age at adiposity peak (AGEAP) on childhood body fat.

## Methods

### Participants

This study was embedded in the Generation R Study, a population-based prospective cohort study from early fetal life onward.^14^ Pregnant women with a delivery date between April 2002 and January 2006, living in Rotterdam, the Netherlands, were eligible for participation. Details on response and follow-up have been described previously.^14^ We had information on fetal or infant growth in 9257 singleton births. Analyses were restricted to a subgroup of 3205 children for whom we had information on visceral or organ fat. The flowchart of participants is given in the eFigure in the Supplement. Written informed consent was provided by the parents for all children. The Medical Ethics Committee of Erasmus Medical Center approved the study. This study followed the Strengthening the Reporting of Observational Studies in Epidemiology (STROBE) reporting guideline.

### Fetal and Infant Growth Measures

As described previously, fetal ultrasound examinations were performed in the first trimester (median, 13.1 weeks [90% range, 11.0-17.0 weeks]), second trimester (median, 20.5 weeks [90% range, 18.9-22.7 weeks]), and third trimester (median, 30.4 weeks [90% range, 28.9-32.3 weeks]).^14^ Of the population under study, 71.4% (2287 of 3205 mothers) had information available for all trimesters. Ultrasound examinations were performed by well-trained staff according to clinical standards. First-trimester ultrasonography was used for establishing gestational age.^15^ In second-trimester and third-trimester ultrasonography, head circumference, abdominal circumference, and femur length were measured to the nearest millimeter. Estimated fetal weight was calculated using the formula by Hadlock et al.^16^ We calculated standard deviation scores (SDS) for estimated fetal weight. Birth weight was obtained from community midwife and hospital registries. We calculated gestational age–adjusted and sex-adjusted SDS for birth weight using World Health Organization fetal growth charts.^17^ Children born SGA were defined as gestational age–adjusted and sex-adjusted SDS for birth weight below the fifth percentile and those born LGA were defined as gestational age–adjusted and sex-adjusted SDS for birth weight above the 95th percentile.

Infant weight was measured in community health centers with a mechanical personal scale around age 6 months (median, 6.2 months [90% range, 5.5-7.5 months]), 12 months (median, 11.1 months [90% range, 10.2-12.3 months]), and 24 months (median, 24.8 months [90% range, 23.6-27.5 months]).^14^ We created age-adjusted and sex-adjusted SDS using Dutch reference growth charts in Growth Analyzer 4.0.^18^

*Fetal weight change* was defined as growth between the second trimester and birth. *Infant weight change* was defined as growth from birth to 24 months (available in 3666 of 5526 children [66.3%]). If weight at 24 months was not available, we used weight at 11 months (available in 746 of 1860 children [40.1%]) and if weight at 11 months was not available, we used weight at 6 months (available in 169 of 1114 children [15.2%]). We considered an increase of more than 0.67 SD between time points as growth acceleration and a decrease of more than 0.67 SD between time points as growth deceleration, reflecting the difference between 2 percentile lines on the growth charts.^19^

Repeated infant measurements were used to derive PWV, AGEAP, and BMIAP, as described previously.^14^ Peak weight velocity in infancy was derived using the Reed1 model for boys and girls separately.^6,20,21,22^ To obtain BMIAP and AGEAP, a cubic mixed-effects model was fitted on log (BMI) from age 2 weeks to 1.5 years, adjusted for sex.^6,21^

### General, Visceral, and Organ Fat

Visceral and organ adiposity were obtained from magnetic resonance imaging scans performed in the brain, thorax, liver, and abdomen, as described previously.^14^ Briefly, all children underwent imaging using a 3.0-T magnetic resonance imaging scanner (Discovery MR750w; GE Healthcare). Pericardial fat imaging in short axis orientation was performed using an electrocardiogram-triggered black-blood–prepared thin-slice single-shot fast-spin echo acquisition with multi-breath-hold approach. An axial 3-point Dixon acquisition for fat and water separation (IDEAL IQ) was used for liver fat imaging.^23^ An axial abdominal scan from lower liver to pelvis and a coronal scan centered at the head of the femurs were performed with a 2-point Dixon acquisition (LavaFlex).

The scans were analyzed by the Precision Image Analysis company, using the sliceOmatic (TomoVision) software package. Extraneous structures and image artifacts were removed manually.^24^ Pericardial fat included both epicardial and paracardial fat directly attached to the pericardium, ranging from the apex to the left ventricular outflow tract. Total visceral fat volume ranged from the dome of the liver to the superior part of the femoral head. Fat mass was obtained by multiplying the total volumes by the specific gravity of adipose tissue, 0.9 g/mL. Liver fat fraction was determined by taking 4 samples of at least 4 cm^2^ from the central portion of the hepatic volume. Subsequently, the mean signal intensities were averaged to generate overall mean liver fat fraction estimation.

At 10 years, we calculated BMI as weight in kilograms divided by height in meters squared, measured without shoes and heavy clothing. Total fat mass and fat-free mass were measured using dual-energy x-ray absorptiometry scanning (iDXA; GE-Lunar), and analyzed with enCORE software, version 12.6.^25^ Children were scanned in the supine position without shoes, heavy clothing, and metal objects, with their hands flat and pronated.

To create adiposity measures independent of height, we estimated optimal adjustment by log-log regression analyses.^26^ All adiposity measures and height were log-transformed, using natural logs. Log-adiposity measures were regressed on log-height. The regression slope corresponds to the power by which height should be raised to calculate an index uncorrelated with height. We divided fat mass by height^4^ (fat mass index [FMI]), fat-free mass by height^2^ (fat-free mass index), and visceral and pericardial fat by height^3^ (visceral and pericardial fat indices).

### Covariates

We obtained information about maternal age, prepregnancy weight, height, parity, educational level, smoking, and folic acid use during pregnancy at enrollment.^14^ Child’s ethnicity was classified by countries of birth of the parents.^27^ Information on duration of breastfeeding was assessed by questionnaires.

### Statistical Analysis

Data analysis was performed from July 26, 2018, to February 7, 2019. First, we performed conditional regression analysis to identify independent critical early-life weight associated with childhood adiposity. Conditional regression analyses take into account correlations between early life growth measures at different ages.^1,28^ We constructed weight variables, statistically independent from weight at earlier time points, using standardized residuals resulting from linear regression models of weight regressed on prior weights.^29^ This approach allows simultaneous inclusion of growth measures in regression models to identify critical growth periods.^29^ We used linear regression analysis to estimate the association of early weight with childhood adiposity, independent from prior weights. Participants were included if they had data available on weight at a specific time point and weight at all prior time points. Second, we categorized fetal and infant weight change into 3 groups (growth deceleration, normal growth, and growth acceleration), and created a combined variable that reflects 9 different growth patterns. We used multivariable linear regression models to assess the associations of combined fetal and infant weight change with childhood adiposity. Finally, we used multivariable linear regression models to assess the associations of PWV, BMIAP, and AGEAP with childhood adiposity. Because we observed statistical interactions between birth size and these growth measures, we stratified the analyses in groups of children born SGA, appropriate for gestational age (AGA), and LGA. No corrections for multiple testing were used as all outcomes represent childhood adiposity. We adjusted basic models for child age and sex. We additionally included covariates based on their association with fat distribution previously or a change in effect size greater than 10% after inclusion in the model. As FMI, visceral fat index, liver fat fraction, and pericardial fat index had skewed distributions, we applied a natural log transformation. We calculated SDS (observed value–mean/SD) for all measures. We performed multiple imputation of missing covariates by generating 5 independent data sets using the Markov Chain Monte Carlo method, presenting pooled effect estimates (95% CI).^30^ Statistical analyses were performed with the SPSS, version 24.0 for Windows (SPSS IBM).

## Results

### Participant Characteristics

The sample consisted of 3205 children (1632 girls and 1573 boys; mean [SD] age, 9.8 [0.3] years) (Table 1). Mothers of children born SGA were more often lower educated than mothers of children born AGA and mothers of children born LGA (102 of 183 [55.7%] vs 1199 of 2514 [47.7%] vs 86 of 210 [41.0%]) and more likely to smoke during pregnancy (65 of 166 [39.2%] vs 481 of 2176 [22.1%] vs 29 of 166 [17.5%]). Children born SGA had the lowest median BMI at 10 years compared with children born AGA and LGA (16.4 [90% range, 14.1-23.6] vs 16.9 [90% range, 14.4-22.8] vs 17.4 [90% range, 14.9-22.7]) (Table 2). Children not included in the analyses were more often non-European compared with children included in the analyses (eTable 1 in the Supplement).

**Table 1.** Participant Characteristics

Characteristic	Participants, No. (%)^a^				*P* Value
	Total Group (N = 3205)	Small for Gestational Age (n = 199)	Appropriate for Gestational Age (n = 2714)	Large for Gestational Age (n = 222)	
Maternal pregnancy characteristics					
Age, mean (SD), y^b^	31.1 (4.9)	30.2 (5.4)	31.1 (4.8)	31.9 (4.4)	<.001
Height, mean (SD), cm^b^	168.0 (7.3)	164.5 (6.9)	168.0 (7.3)	171.6 (6.9)	<.001
Weight, median (90% range), kg^b^	64.0 (50.0-90.0)	60.0 (47.4-76.7)	64.0 (50.0-90.0)	70.0 (57.0-97.0)	<.001
Prepregnancy body mass index, median (90% range)^b^^,^^c^	22.5 (18.7-32.0)	22.2 (17.9-29.4)	22.4 (18.6-32.0)	24.0 (20.1-32.4)	<.001
Parity^d^					
0	1787/3091 (57.8)	155/197 (78.7)	1506/2634 (57.2)	90/212 (42.5)	<.001
≥1	1304/3091 (42.2)	42/197 (21.3)	1128/2634 (42.8)	122/212 (57.5)	
Educational level^d^					
Lower	1400/2954 (47.4)	102/183 (55.7)	1199/2514 (47.7)	86/210 (41.0)	.01
Higher	1554/2954 (52.6)	81/183 (44.3)	1315/2514 (52.3)	124/210 (59.0)	
Folic acid use^d^					
No	445/2204 (20.2)	35/137 (25.5)	384/1893 (20.3)	20/142 (14.1)	.06
Yes	1759/2204 (79.8)	102/137 (74.5)	1509/1893 (79.7)	122/142 (85.9)	
Smoking during pregnancy^d^					
No	1968/2550 (77.2)	101/166 (60.8)	1695/2176 (77.9)	137/166 (82.5)	<.001
Yes	582/2550 (22.8)	65/166 (39.2)	481/2176 (22.1)	29/166 (17.5)	
Birth and infant characteristics					
Boys^d^	1573 (49.1)	100 (50.3)	1347 (49.6)	86 (38.7)	.01
Birth weight, mean (SD), g^b^	3444 (554)	2471 (427)	3436 (439)	4322 (437)	<.001
Race/ethnicity^d^					
European	2134/3141 (67.9)	112/194 (57.7)	1797/2663 (67.5)	177/219 (80.8)	<.001
Non-European	1007/3141 (32.1)	82/194 (42.3)	866/2663 (32.5)	42/219 (19.2)	
Breastfeeding, ever^d^	2513/2708 (92.8)	142/156 (91.0)	2151/2313 (93.0)	179/194 (92.3)	.62

^a^ Characteristics are based on observed, not imputed, data.

^b^ Differences in characteristics for children born small for gestational age, appropriate for gestational age, and large for gestational age were evaluated using 1-way analysis of variance for continuous variables.

^c^ Calculated as weight in kilograms divided by height in meters squared.

^d^ Differences in characteristics for children born small for gestational age, appropriate for gestational age, and large for gestational age were evaluated using χ^2^ test for categorical variables.

**Table 2.** Fetal, Infant, and Childhood Anthropometrics

Characteristic	Median (90% Range)^a^				*P* Value
	Total Group (N = 3205)	Small for Gestational Age (n = 199)	Appropriate for Gestational Age (n = 2714)	Large for Gestational Age (n = 222)	
**Fetal Characteristics**					
Second trimester, mean (SD)^b^					
Gestational age, wk	20.6 (1.1)	20.5 (1.1)	20.6 (1.1)	20.6 (1.1)	.75
Estimated fetal weight, g	378 (89)	353 (79)	378 (89)	399 (92)	<.001
Third trimester, mean (SD)^b^					
Gestational age, wk	30.4 (1.0)	30.3 (1.1)	30.4 (1.0)	30.4 (0.9)	.25
Estimated fetal weight, g	1622 (249)	1403 (216)	1623 (239)	1816 (246)	<.001
Birth characteristics					
Gestational age, wk^b^	40.1 (37.0-42.0)	39.2 (33.0-41.7)	40.1 (37.1-42.0)	40.3 (36.4-42.0)	<.001
Length, mean (SD), cm^b^	50.3 (2.3)	47.2 (1.9)	50.3 (2.1)	52.9 (2.1)	<.001
Weight, mean (SD), g^b^	3444 (554)	2471 (427)	3436 (439)	4322 (437)	<.001
Preterm birth (<37 wk), No. (%)^c^	149/3182 (4.7)	28 (14.1)	106 (3.9)	13 (5.9)	<.001
Low birth weight (<2500 g), No. (%)^c^	128 (4.0)	72 (36.2)	52 (1.9)	3 (1.4)	<.001
**Infant Characteristics**					
At 6 mo^b^					
Age at visit, mo	6.2 (5.4-7.2)	6.2 (5.5-6.9)	6.2 (5.5-7.1)	6.2 (5.4-7.7)	.75
Length, cm	67.5 (63.0-72.0)	65.0 (60.8-69.6)	67.3 (64.0-71.6)	69.0 (65.0-73.0)	<.001
Weight, kg	7.8 (6.5-9.4)	7.0 (5.7-8.4)	7.8 (6.5-9.4)	8.2 (6.8-9.7)	<.001
At 12 mo^b^					
Age at visit, mo	11.0 (10.2-12.3)	11.0 (10.2-12.3)	11.0 (10.2-12.3)	11.1 (10.2-12.5)	.57
Length, cm	74.0 (70.0-78.5)	72.5 (67.5-76.9)	74.0 (70.1-78.5)	75.5 (71.1-79.1)	<.001
Weight, kg	9.6 (8.0-11.4)	8.7 (7.2-10.4)	9.6 (8.0-11.4)	10.1 (8.7-11.7)	<.001
At 2 y^b^					
Age at visit, y	2.1 (2.0-2.3)	2.0 (2.0-2.2)	2.1 (2.0-2.3)	2.1 (2.0-2.3)	.03
Length, cm	88.0 (82.5-94.0)	86.0 (81.0-92.1)	88.0 (83.0-94.0)	90.0 (84.2-95.1)	<.001
Weight, kg	12.8 (10.7-15.6)	11.6 (9.8-14.0)	12.8 (10.8-15.5)	13.7 (11.4-16.2)	<.001
PWV, mean (SD), kg/y^b^	12.1 (2.1)	12.2 (2.1)	12.2 (2.1)	11.4 (2.0)	<.001
BMIAP, mean (SD)^b^^,^^d^	17.6 (0.8)	17.0 (0.7)	17.6 (0.8)	18.0 (0.8)	<.001
AGEAP, mo^b^	8.4 (7.8-9.6)	9.0 (7.8-9.6)	8.4 (7.8-9.6)	8.4 (7.8-9.6)	<.001
Childhood characteristics					
Age at visit, mean (SD), y^b^	9.8 (0.3)	9.8 (0.3)	9.8 (0.3)	9.8 (0.4)	.65
Height, mean (SD), cm^b^	141.7 (6.7)	138.8 (6.4)	141.6 (6.6)	144.9 (6.7)	<.001
Weight, kg^b^	34.0 (26.0-49.0)	31.0 (24.8-49.0)	33.8 (26.6-49.0)	36.8 (28.4-51.8)	<.001
Body mass index^b^^,^^d^	16.9 (14.4-22.9)	16.4 (14.1-23.6)	16.9 (14.4-22.8)	17.4 (14.9-22.7)	<.001
Overweight or obese, No. (%)^c^	556 (17.4)	31 (15.6)	463 (17.1)	50 (22.5)	<.001
Fat mass index, kg/m^4^^b^	2.1 (1.3-4.3)	2.2 (1.3-4.5)	2.1 (1.3-4.3)	2.1 (1.3-4.1)	.67
Fat-free mass index^b^^,^^d^	12.5 (11.0-14.5)	12.3 (10.7-14.1)	12.5 (11.0-14.4)	12.8 (11.4-14.8)	<.001
Visceral adipose tissue, g^b^	358 (182-834)	348 (156-802)	357 (184-843)	368 (189-849)	.38
Liver fat, %^b^	2.0 (1.3-4.1)	2.1 (1.3-4.6)	2.0 (1.3-4.1)	2.0 (1.2-3.9)	.79
Pericardial fat, g^b^	10.6 (5.3-20.3)	9.5 (4.5-18.7)	10.6 (5.3-20.4)	11.5 (6.0-20.9)	<.001

Abbreviations: AGEAP, age at adiposity peak; BMIAP, body mass index at adiposity peak; PWV, peak weight velocity.

^a^ Characteristics are based on observed, not imputed, data.

^b^ Differences in characteristics for children born small for gestational age, appropriate for gestational age and large for gestational age were evaluated using 1-way analysis of variance for continuous variables.

^c^ Differences in characteristics for children born small for gestational age, appropriate for gestational age and large for gestational age were evaluated using χ^2^ test for categorical variables.

^d^ Calculated as weight in kilograms divided by height in meters squared.

### Critical Periods During Fetal and Infant Growth

Table 3 shows the difference in SDS visceral and organ fat outcomes per 1-SDS change in fetal and infant weight, independent from prior weights based on conditional models. A change of 1 SDS in third trimester weight was, independent from second trimester weight, positively associated with pericardial fat index (SDS, 0.07; 95% CI, 0.01-0.12) and inversely associated with liver fat fraction (SDS, –0.06; 95% CI, –0.11 to –0.01). A 1-SDS change in birth weight was positively associated with pericardial fat index (SDS, 0.06; 95% CI, 0.01-0.12). Higher infant weight at 6, 12, and 24 months was associated with higher visceral fat (6 months: SDS, 0.07; 95% CI, 0.01 to 0.12; 12 months: SDS, 0.07; 95% CI, 0.02 to 0.12; and 24 months: SDS, 0.05; 95% CI, –0.001 to 0.10) and liver fat fraction (6 months: SDS, 0.06; 95% CI, 0.01-0.11; 12 months: SDS, 0.07; 95% CI, 0.02-0.12; and 24 months: SDS, 0.06; 95% CI, 0.01-0.11). Effect estimates for infant weight tended to be larger than those for fetal and birth weight, although the 95% CIs overlap. eTable 2 in the Supplement shows positive associations of third trimester weight, birth weight, and infant weight with measures of general fat. Results from basic models showed similar results (eTable 3 in the Supplement).

**Table 3.** Associations of Fetal and Infant Growth With Childhood Adiposity Fat From Conditional Analyses

Infant and Fetal Weight Standard Deviation Scores	Standard Deviation Scores, Regression Coefficients (95% CI)^a^		
	Visceral Fat Index (n = 2731)	Liver Fat Fraction (n = 3058)	Pericardial Fat Index (n = 2839)
At 20 wk (n = 2729)	−0.02 (−0.07 to 0.04)	0.03 (−0.02 to 0.08)	0.01 (−0.05 to 0.06)
At 30 wk (n = 2676)	−0.01 (−0.07 to 0.04)	−0.06 (−0.11 to −0.01)	0.07 (0.01 to 0.12)
At birth (n = 2676)	−0.01 (−0.06 to 0.05)	−0.02 (−0.07 to 0.03)	0.06 (0.01 to 0.12)
At 6 mo (n = 2212)	0.07 (0.01 to 0.12)	0.06 (0.01 to 0.11)	0.04 (−0.02 to 0.09)
At 12 mo (n = 1912)	0.07 (0.02 to 0.12)	0.07 (0.02 to 0.12)	0.01 (−0.05 to 0.06)
At 24 mo (n = 1539)	0.05 (−0.001 to 0.10)	0.06 (0.01 to 0.11)	−0.004 (−0.06 to 0.05)

^a^ Regression coefficients are linear regression coefficients from conditional analyses based on standard deviation scores of natural log–transformed outcome measures. Models are adjusted for family-based sociodemographic factors (maternal age and educational level), maternal lifestyle-related factors (prepregnancy body mass index, smoking during pregnancy, folic acid use during pregnancy, and parity), and childhood factors (age at visit, sex, race/ethnicity, and breastfeeding).

### Associations With Fetal and Infant Growth Patterns

We examined 9 different growth patterns and observed that, compared with children with normal fetal and infant growth (533 of 2370 [22.5%]), those with fetal weight deceleration followed by infant weight acceleration (263 of 2370 [11.1%]) had the highest visceral fat index (SDS, 0.18; 95% CI, 0.03-0.33; *P* = .02) and liver fat fraction (SDS, 0.34; 95% CI, 0.20-0.48; *P* < .001) (Table 4). eTable 4 in the Supplement shows the highest general fat in children with both fetal and infant weight acceleration (116 of 2370 [4.9%]). In eTable 5 in the Supplement, medians and 90% ranges of visceral and organ fat measures were shown in their original unit for all 9 growth patterns, showing the highest median visceral fat and liver fat fraction in children with fetal growth deceleration and infant growth acceleration. A statistically significant interaction between fetal and infant growth was observed for BMI, FMI, and liver fat fraction (Table 4; eTable 6 in the Supplement). Results from basic models showed similar results (eTable 6 in the Supplement).

**Table 4.** Associations of Fetal and Infant Growth With Childhood General, Visceral, and Organ Fat

Characteristic	Standard Deviation Scores, Regression Coefficients (95% CI)^a^		
	Visceral Fat Index (n = 2731)	Liver Fat Fraction (n = 3058)	Pericardial Fat Index (n = 2839)
Fetal growth deceleration			
Infant growth deceleration (n = 78)	−0.01 (−0.26 to 0.23)	−0.06 (−0.29 to 0.17)	−0.07 (−0.31 to 0.18)
Infant normal growth (n = 261)	−0.22 (−0.37 to −0.06)	−0.06 (−0.21 to 0.08)	−0.08 (−0.23 to 0.07)
Infant growth acceleration (n = 263)	0.18 (0.03 to 0.33)	0.34 (0.20 to 0.48)	−0.09 (−0.24 to 0.07)
Fetal normal growth			
Infant growth deceleration (n = 213)	−0.08 (−0.24 to 0.08)	−0.11 (−0.27 to 0.04)	0.05 (−0.12 to 0.22)
Infant normal growth (n = 533)	[Reference]	[Reference]	[Reference]
Infant growth acceleration (n = 271)	0.07 (−0.08 to 0.22)	0.09 (−0.05 to 0.23)	−0.07 (−0.23 to 0.08)
Fetal growth acceleration			
Infant growth deceleration (n = 319)	−0.08 (−0.22 to 0.07)	−0.06 (−0.20 to 0.07)	0.15 (0.01 to 0.30)
Infant normal growth (n = 316)	0.08 (−0.06 to 0.22)	0.03 (−0.10 to 0.17)	0.08 (−0.06 to 0.23)
Infant growth acceleration (n = 116)	0.05 (−0.16 to 0.25)	−0.08 (−0.27 to 0.12)	0.08 (−0.13 to 0.29)
*P* value for interaction	.09	<.001	.45

^a^ Regression coefficients are linear regression coefficients based on standard deviation scores of natural log–transformed outcome measures. Models are adjusted for family-based sociodemographic factors (maternal age and educational level), maternal lifestyle-related factors (prepregnancy body mass index, smoking during pregnancy, folic acid use during pregnancy, and parity), and childhood factors (age at visit, sex, race/ethnicity, and breastfeeding). *P* value of interaction term is between fetal and infant growth in model adjusted for childhood age at visit and sex.

### Size at Birth and Longitudinal Infant Growth Patterns

Table 5 shows associations of a higher PWV and BMIAP with higher visceral fat index and liver fat fraction in children born SGA and AGA and with visceral fat index in children born LGA. Results for measures of general fat and from basic models are shown in eTable 7 and eTable 8 in the Supplement.

**Table 5.** Associations of Infant Growth Patterns With Childhood General, Visceral, and Organ Fat

Characteristic	Standard Deviation Scores, Regression Coefficients (95% CI)^a^		
	Visceral Fat Index (n = 2731)	Liver Fat Fraction (n = 3058)	Pericardial Fat Index (n = 2839)
Children born small for gestational age			
PWV, kg/y (n = 161)	0.10 (−0.01 to 0.21)	0.08 (−0.02 to 0.14)	0.04 (−0.07 to 0.14)
BMIAP (n = 151)	0.13 (−0.15 to 0.41)	0.05 (−0.18 to 0.28)	0.08 (−0.17 to 0.34)
AGEAP, mo (n = 151)	0.31 (−0.05 to 0.67)	0.16 (−0.14 to 0.47)	0.06 (−0.28 to 0.40)
Children born appropriate for gestational age			
PWV, kg/y (n = 2352)	0.04 (0.02 to 0.06)	0.04 (0.02 to 0.06)	0.02 (−0.001 to 0.04)
BMIAP (n = 2207)	0.19 (0.13 to 0.25)	0.14 (0.08 to 0.20)	0.11 (0.05 to 0.16)
AGEAP, mo (n = 2207)	−0.01 (−0.09 to 0.08)	−0.03 (0.11 to 0.05)	−0.002 (−0.09 to 0.09)
Children born large for gestational age			
PWV, kg/y (n = 195)	0.06 (−0.02 to 0.13)	0.06 (−0.01 to 0.14)	0.003 (−0.07 to 0.08)
BMIAP (n = 185)	−0.01 (−0.19 to 0.18)	0.05 (−0.15 to 0.24)	0.03 (−0.17 to 0.22)
AGEAP, mo (n = 185)	−0.04 (−0.29 to 0.21)	0.13 (−0.15 to 0.40)	0.06 (−0.21 to 0.34)

Abbreviations: AGEAP, age at adiposity peak; BMIAP, body mass index at adiposity peak (calculated as weight in kilograms divided by height in meters squared); PWV, peak weight velocity.

^a^ Regression coefficients are linear regression coefficients based on standard deviation scores of natural log–transformed outcome measures. Models are adjusted for family-based sociodemographic factors (maternal age and educational level), maternal lifestyle-related factors (prepregnancy body mass index, smoking during pregnancy, folic acid use during pregnancy, and parity), and childhood factors (age at visit, sex, race/ethnicity, birth weight, and breastfeeding).

## Discussion

In this large population-based prospective cohort study in the Netherlands, we observed that fetal life and, especially, infancy seem to be independent critical periods for the development of childhood adiposity. In addition, children with fetal weight deceleration followed by infant weight acceleration had the highest visceral and liver fat. Finally, a higher PWV and BMIAP were associated with an adverse childhood visceral and organ fat distribution.

### Interpretation of Main Findings

Early-life growth influences the risk of overweight later in life.^1,3,31,32,33^ As fetal and infant growth are correlated, it is important to study their independent associations with childhood adiposity. A previous study reported that infant weight was positively associated with BMI, FMI, android to gynoid ratio, and abdominal fat at age 6 years, independently from prior weight.^1^ Third trimester weight and birth weight were positively associated with childhood BMI only. Larger effect estimates were present for infant weight. We extended that study by performing a longer-term follow-up study and by studying more detailed measures of organ fat at age 10 years. Although excess visceral fat and organ fat have been associated with increased risk of adverse cardiometabolic phenotypes, little is known about their early determinants.^10,11,12^ To our knowledge, this study is the first to report associations of early growth with visceral and organ fat.

We observed that, independent from prior weight, third trimester fetal weight and birth weight were positively associated with measures of general fat and pericardial fat index at age 10 years. Infant weight was positively associated with general, visceral, and liver fat. A tendency of larger effect estimates was observed for infant weight. Results of a previous population-based study among 2453 Swedish adolescents suggested that birth weight and weight at 1 year, adjusted for birth weight, were positively associated with BMI, FMI, fat-free mass index by height, waist circumference, and fat percentage at age 15 years, with larger effect estimates present for infant weight.^34^ Positive associations of infant weight, independent from birth weight, with childhood adiposity were also suggested by a population-based prospective cohort study among 561 UK participants aged 7 years.^35^ Thus, results from our study and from previous literature suggest that both fetal and infant growth influence childhood adiposity. A tendency of larger effect estimates for infant weight indicate that infancy might be a more important critical period for the development of adiposity and organ fat.

A recent review including 18 cohort studies reported that the risk of childhood obesity is increased with rapid postnatal growth across the full range of birth weights.^31^ In addition, a previous study reported that, compared with children with normal fetal and infant growth, those with fetal growth deceleration followed by infant growth acceleration had a higher FMI, android to gynoid ratio, and abdominal fat at age 6 years.^1^ Another study among 6075 Chinese children examined the interaction between birth weight and infant weight gain from birth to 3 months. Compared with children with a low birth weight and infant growth deceleration, children of all birth weights with infant growth acceleration had a higher BMI at age 7 years.^36^ Next to their independent associations, we examined the combined associations of fetal and infant growth with childhood adiposity. We observed that children with both fetal and infant weight acceleration had higher general fat measures. However, only a small group of children showed fetal and infant weight acceleration, as infant weight acceleration is more common in children with fetal weight deceleration.^2,4,5,31^ Children with fetal weight deceleration followed by infant weight acceleration had the highest visceral fat index and liver fat fraction. From these findings, we can conclude that rapid postnatal growth seems to lead to higher childhood adiposity in children with different fetal weight gain patterns. Children who experienced fetal growth restriction but show postnatal catch-up growth may be at a higher risk for visceral fat accumulation and liver steatosis.

We used longitudinal early-life growth data to derive PWV, BMIAP, and AGEAP. A higher PWV and BMIAP were associated with higher general, visceral, and organ fat in children born SGA, AGA, and LGA. These findings are in line with those of previous studies from the same cohort at younger ages, showing positive associations of PWV and BMIAP with BMI, body fat percentage, android to gynoid ratio, and abdominal fat at ages 4 and 6 years.^6,7^ Our findings are also in line with those of previous studies reporting positive associations of BMIAP with fat mass among 311 Danish children aged 3 years^8^ and with waist circumference in 4121 Finnish adults.^9^ Our findings suggest that, next to its association with obesity and measures of general and abdominal adiposity, early growth weight velocity patterns are also associated with childhood visceral and organ fat at age 10 years.

Our findings emphasize the importance of early growth patterns in association with the development of childhood visceral and organ fat. Although the observed effects were small to moderate, they seem relevant because obesity tends to track into adulthood. Also, even subclinical differences in visceral and organ fat are associated with the development of cardiovascular diseases in later life. Future studies should determine optimal growth in association with later health for use in clinical practice. Whether and which nutritional or other factors influence growth and lead to increases in pericardial and liver fat needs to be studied further. Future studies are also needed to determine potential strategies to intervene in order to prevent later obesity and related health complications in infants who have had fetal growth deceleration and show infant growth acceleration.

### Methodological Considerations

This study had detailed information available on early growth and childhood adiposity. Magnetic resonance imaging is considered the criterion standard for measures of visceral and organ fat.^24,37,38,39^ We have used IDEAL IQ for liver fat quantification, rather than magnetic resonance spectroscopy, because IDEAL IQ acquires an image of the entire liver in a single breath-hold. In addition, IDEAL IQ automatically processes the liver fat fraction. Therefore, IDEAL IQ is adequate to use in large population-based studies.

### Limitations

Estimation of fetal weight by ultrasonography might be prone to measurement error, especially among fetuses with low or high weight, which might have influenced our findings.^40^ Of the 9257 singleton live births with information on fetal or infant growth, 3205 children had information on visceral and organ fat at age 10 years. Children included in the analysis were more frequently breastfed compared with children who were not included and their mothers more often had a higher educational level. These factors might have affected the generalizability of our results. In case of selection bias, associations of early growth with childhood adiposity would differ between these groups.^41^ For defining infant weight change, we considered the period from birth to 24 months. When weight information was missing at 24 months, we used weight at 11 months or 6 months. This approach allowed an adequate sample size and statistical power on subgroup analyses, but might have introduced bias. Although we adjusted for a large number of potential confounders, residual confounding might still be an issue. As an example, information on pubertal status was not available.

## Conclusions

Our findings suggest that early-life weight, in particular infant weight, is associated with future visceral and organ fat accumulation. Fetal growth restriction followed by infant growth acceleration is associated with increased visceral and liver fat. Further studies are needed to examine long-term health consequences of different early growth patterns.

## Notes

Supplement. **eTable 1.** Nonresponse Analysis in Singleton Live Births With and Without Measures of Adiposity Available **eTable 2.** Associations of Fetal and Infant Growth With Childhood Body Composition From Conditional Analyses **eTable 3.** Associations of Fetal and Infant Growth With Childhood Body Composition and Adiposity From Conditional Analyses **eTable 4.** Associations of Fetal and Infant Growth With Childhood Body Composition **eTable 5.** Visceral and Organ Fat for 9 Different Growth Patterns **eTable 6.** Associations of Fetal and Infant Growth With Childhood Body Composition and Adiposity **eTable 7.** Associations of Infant Growth Patterns With Childhood Body Composition **eTable 8.** Associations of Infant Growth Patterns With Childhood Body Composition and Adiposity **eFigure.** Flowchart of Participants
