# Supplementary material for: Associations of Fetal and Infant Weight Change With General, Visceral, and Organ Adiposity at School Age
Source: JAMA Netw Open. 2019 Apr 26;2(4):e192843. doi: 10.1001/jamanetworkopen.2019.2843 (PMC6487630; doi:10.1001/jamanetworkopen.2019.2843)
Supplement: Supplement. — eTable 1. Nonresponse Analysis in Singleton Live Births With and Without Measures of Adiposity Available eTable 2. Associations of Fetal and Infant Growth With Childhood Body Composition From Conditional Analyses eTable 3. Associations of Fetal and Infant Growth With Childhood Body Composition and Adiposity From Conditional Analyses eTable 4. Associations of Fetal and Infant Growth With Childhood Body Composition eTable 5. Visceral and Organ Fat for 9 Different Growth Patterns eTable 6. Associations of Fetal and Infant Growth With Childhood Body Composition and Adiposity eTable 7. Associations of Infant Growth Patterns With Childhood Body Composition eTable 8. Associations of Infant Growth Patterns With Childhood Body Composition and Adiposity eFigure. Flowchart of Participants [file jamanetwopen-2-e192843-s001.pdf]

Supplementary Online Content

Vogelezang S, Santos S, Toemen L, Oei EHG, Felix JF, Jaddoe VWV. Associations of fetal and infant weight change with general, visceral, and organ adiposity at school age. *JAMA Netw Open.* 2019;2(4):e192843. doi:10.1001/jamanetworkopen.2019.2843

eTable 1. Nonresponse Analysis in Singleton Live Births With and Without Measures of Adiposity Available
eTable 2. Associations of Fetal and Infant Growth With Childhood Body Composition From Conditional Analyses
eTable 3. Associations of Fetal and Infant Growth With Childhood Body Composition and Adiposity From Conditional Analyses
eTable 4. Associations of Fetal and Infant Growth With Childhood Body Composition
eTable 5. Visceral and Organ Fat for 9 Different Growth Patterns
eTable 6. Associations of Fetal and Infant Growth With Childhood Body Composition and Adiposity
eTable 7. Associations of Infant Growth Patterns With Childhood Body Composition
eTable 8. Associations of Infant Growth Patterns With Childhood Body Composition and Adiposity
eFigure. Flowchart of Participants

This supplementary material has been provided by the authors to give readers additional information about their work.

© 2019 Vogelezang S et al. *JAMA Network Open.*

eTable 1. Nonresponse Analysis in Singleton Live Births With and Without Measures of Adiposity Available

**Characteristics**	**Participants, No (%)**[a]		
	**Children included in the analysis (n= 3205)**	**Children not included in the analysis (n= 6696)**	***P* Value**
**Maternal pregnancy characteristics**			
Age (years), mean (SD)[b]	31.1 (4.9)	29.4 (5.5)	**<.001**
Height (cm), mean (SD)[b]	168.0 (7.3)	166.8 (7.4)	**<.001**
Weight (kg), median (90% range)[b]	64.0 (50.0; 90.0)	64.0 (50.0; 91.0)	.28
Pre-pregnancy body mass index, median (90% range)[b,c]	22.5 (18.7; 32.0)	22.7 (18.6; 33.8)	.06
Parity[d]			**<.001**
0	1787/3091 (57.8)	3449/6426 (53.7)	
≥ 1	1304/3091 (42.4)	2977/6426 (46.3)	
Educational level[d]			**<.001**
Lower	1400/2954 (47.4)	3546/5704 (62.2)	
Higher	1554/2954 (52.6)	2158/5704 (37.8)	
Folic acid use[d]			**<.001**
No	445/2204 (20.2)	1498/4427 (33.8)	
Yes	1759/2204 (79.8)	2929/4427 (66.2)	
Smoking during pregnancy[d]			**<.001**
No	1968/2550 (77.2)	3717/5272 (70.5)	
Yes	582/2550 (22.8)	1555/5272 (29.5)	
**Childhood characteristics**			
Boys[d]	1573/3205 (49.1)	3359/6536 (51.4)	**.03**
Birth weight (g), mean (SD)[b]	3444 (554)	3356 (600)	**<.001**
Age at visit (years), mean (SD)[b]	9.8 (0.3)	9.8 (0.4)	**<.001**
Race/ethnicity[d]			**<.001**
European	2134/3141 (67.0)	3550/6076 (58.4)	
Non-European	1007/3141 (32.1)	2526/6076 (41.6)	
Breastfeeding, ever[d]	2513 (92.8)	3708 (91.1)	**.01**

[a]Characteristics are based on observed, not imputed data.
[b]Differences in characteristics for children with and without fat measures available were evaluated using Independent-samples T test for continuous variables.
[c]Calculated as weight in kilograms divided by height in meters squared.
[d]Differences in characteristics for children with and without fat measures available were evaluated using χ2 test for categorical variables.

© 2019 Vogelezang S et al. *JAMA Network Open.*

eTable 2. Associations of Fetal and Infant Growth With Childhood Body Composition From Conditional Analyses

Infant and Fetal Weight Standard Deviation Scores	Standard Deviation Scores, Regression Coefficients (95% CI)		
	Body mass index[a,b] (n= 3205)	Fat mass index[b,c] (n= 3185)	Fat free mass index[a,b] (n= 3185)
At 20 wk (n= 2729)	0.04 (0.001; 0.09)	-0.002 (-0.05; 0.04)	0.05 (0.003; 0.09)
At 30 wk (n= 2676)	0.05 (0.01; 0.09)	-0.002 (-0.05; 0.04)	0.05 (0.01; 0.09)
At birth (n= 2676)	0.08 (0.03; 0.12)	-0.01 (-0.05; 0.04)	0.15 (0.11; 0.10)
At 6 mo (n= 2212)	0.20 (0.16; 0.25)	0.11 (0.07; 0.16)	0.19 (0.15; 0.23)
At 12 mo (n= 1912)	0.19 (0.15; 0.23)	0.09 (0.05; 0.14)	0.17 (0.13; 0.21)
At 24 mo (n= 1539)	0.22 (0.18; 0.26)	0.10 (0.05; 0.14)	0.21 (0.17; 0.25)

[a]Coefficients are linear regression coefficients from conditional analyses based on SD scores of the outcome measures.
[b]Models are adjusted for family-based sociodemographic factors (maternal age and educational level), maternal lifestyle-related factors (pre-pregnancy body mass index, smoking during pregnancy, folic acid use during pregnancy, and parity), and childhood factors (age at visit, sex, race/ethnicity, and breastfeeding).
[c]Regression coefficients are linear regression coefficients from conditional analyses based on SD scores of natural log-transformed outcome measures.

© 2019 Vogelezang S et al. *JAMA Network Open.*

eTable 3. Associations of Fetal and Infant Growth With Childhood Body Composition and Adiposity From Conditional Analyses

Infant and Fetal Weight Standard Deviation Scores	Standard Deviation Scores, Regression Coefficients (95% CI)					
	Body mass index[a,b] (n= 3205)	Fat mass index[b,c] (n= 3185)	Fat free mass index[a,b] (n= 3185)	Visceral fat index[b,c] (n= 2731)	Liver fat fraction[b,b] (n= 3058)	Pericardial fat index[b,c] (n= 2839)
At 20 wk (n= 2729)	0.04 (-0.001; 0.09)	0.0004 (-0.05; 0.05)	0.05 (0.004; 0.09)	0.004 (-0.05; 0.06)	0.03 (-0.02; 0.09)	0.01 (-0.04; 0.07)
At 30 wk (n= 2676)	0.05 (0.003; 0.09)	-0.004 (-0.05; 0.04)	0.05 (0.01; 0.09)	-0.003 (-0.06; 0.05)	-0.05 (-0.10; -0.003)	0.07 (0.02; 0.13)
At birth (n= 2676)	0.04 (-0.001; 0.09)	-0.04 (-0.09; 0.004)	0.14 (0.09; 0.18)	-0.01 (-0.06; 0.05)	-0.03 (-0.08; 0.02)	0.06 (0.01; 0.12)
At 6 mo (n= 2212)	0.25 (0.20; 0.29)	0.16 (0.12; 0.21)	0.22 (0.17; 0.26)	0.09 (0.04; 0.14)	0.08 (0.03; 0.13)	0.04 (-0.01; 0.10)
At 12 mo (n= 1912)	0.18 (0.14; 0.23)	0.09 (0.05; 0.14)	0.17 (0.13; 0.21)	0.09 (0.03; 0.14)	0.07 (0.02; 0.12)	0.03 (-0.03; 0.08)
At 24 mo (n= 1539)	0.22 (0.18; 0.27)	0.10 (0.06; 0.15)	0.22 (0.18; 0.26)	0.06 (0.004; 0.11)	0.06 (0.01; 0.11)	0.004 (-0.05; 0.06)

[a]Regression coefficients are linear regression coefficients from conditional analyses based on SD scores of the outcome measures.
[b]Models are adjusted for childhood age at visit and sex.
[c]Regression coefficients are linear regression coefficients from conditional analyses based on SD scores of natural log-transformed outcome measures.

© 2019 Vogelezang S et al. *JAMA Network Open.*

eTable 4. Associations of Fetal and Infant Growth With Childhood Body Composition

Fetal Growth	Infant Growth	Standard Deviation Scores, Regression Coefficients (95% CI)		
		**Body mass index[a,b] (n= 3205)**	**Fat mass index[b,c] (n= 3185)**	**Fat free mass index[a,b] (n= 3185)**
Deceleration	Deceleration (n= 78)	-0.32 (-0.53; -0.12)	-0.17 (-0.37; 0.02)	-0.22 (-0.43; -0.02)
	Normal (n= 261)	-0.16 (-0.29; -0.03)	-0.10 (-0.23; 0.02)	-0.12 (-0.25; 0.01)
	Acceleration (n= 263)	0.27 (0.14; 0.40)	0.19 (0.07; 0.32)	0.20 (0.07; 0.33)
Normal	Deceleration (n= 213)	-0.24 (-0.38; -0.10)	-0.18 (-0.31; -0.04)	-0.17 (-0.30; -0.03)
	Normal (n= 533)	Reference	Reference	Reference
	Acceleration (n= 271)	0.40 (0.27; 0.53)	0.19 (0.07; 0.32)	0.39 (0.26; 0.52)
Acceleration	Deceleration (n= 319)	-0.07 (-0.20; 0.05)	-0.09 (-0.21; 0.03)	0.07 (-0.05; 0.19)
	Normal (n= 316)	0.24 (0.12; 0.36)	0.09 (-0.03; 0.21)	0.26 (0.14; 0.38)
	Acceleration (n= 116)	0.42 (0.24; 0.59)	0.13 (-0.04; 0.30)	0.52 (0.34; 0.70)

[a]Regression coefficients are linear regression coefficients based on SD scores of the outcome.
[b]Models are adjusted for family-based sociodemographic factors (maternal age and educational level), maternal lifestyle-related factors (pre-pregnancy body mass index, smoking during pregnancy, folic acid use during pregnancy, and parity), and childhood factors (age at visit, sex, ethnicity/race, and breastfeeding).
[c]Regression coefficients are linear regression coefficients based on SD scores of natural log-transformed outcome measures.

© 2019 Vogelezang S et al. *JAMA Network Open.*

eTable 5. Visceral and Organ Fat for 9 Different Growth Patterns

Fetal Growth	Infant Growth	Medians (90% range)		
		Visceral fat (g) (n= 2731)	Liver fat fraction (%) (n= 3058)	Pericardial fat (g)[a] (n= 2839)
Deceleration	Deceleration (n= 78)	307 (177; 714)	1.9 (1.3; 4.9)	9.9 (4.1; 15.5)
	Normal (n= 261)	330 (155; 701)	1.9 (1.3; 4.4)	9.9 (5.1; 18.7)
	Acceleration (n= 263)	412 (190; 957)	2.2 (1.4; 5.3)	10.2 (5.2; 21.0)
Normal	Deceleration (n= 213)	338 (187; 694)	1.9 (1.3; 3.4)	10.1 (5.0; 21.2)
	Normal (n= 533)	365 (179; 803)	2.0 (1.3; 3.8)	10.4 (5.1; 20.1)
	Acceleration (n= 271)	398 (207; 956)	2.1 (1.4; 4.3)	11.3 (5.0; 20.6)
Acceleration	Deceleration (n= 319)	348 (183; 805)	1.9 (1.3; 3.8)	10.7 (5.5; 20.1)
	Normal (n= 316)	396 (213; 854)	2.0 (1.4; 3.6)	11.6 (5.9; 21.4)
	Acceleration (n= 116)	411 (209; 982)	2.0 (1.3; 4.0)	11.7 (6.3; 23.4)

© 2019 Vogelezang S et al. *JAMA Network Open.*

eTable 6. Associations of Fetal and Infant Growth With Childhood Body Composition and Adiposity

		Standard Deviation Scores, Regression Coefficients (95% CI)					
**Fetal Growth**	**Infant Growth**	**Body mass index[a,b] (n= 3205)**	**Fat mass index[b,c] (n= 3185)**	**Fat free mass index[a,b] (n= 3185)**	**Visceral fat index[b,c] (n= 2731)**	**Liver fat fraction[b,c] (n= 3058)**	**Pericardial fat index[b,c] (n= 2839)**
Deceleration	Deceleration (n= 78)	-0.30 (-0.52; -0.08)	-0.16 (-0.37; 0.05)	-0.22 (-0.43; -0.01)	0.04 (-0.22; 0.29)	-0.04 (-0.27; 0.19)	-0.04 (-0.28; 0.21)
	Normal (n= 261)	-0.09 (-0.23; 0.05)	-0.02 (-0.15; 0.12)	-0.10 (-0.23; -0.04)	-0.15 (-0.30; 0.01)	-0.01 (-0.16; 0.13)	-0.04 (-0.19; 0.11)
	Acceleration (n= 263)	0.39 (0.25; 0.53)	0.32 (0.19; 0.46)	0.26 (0.12; 0.39)	0.26 (0.10; 0.41)	0.41 (0.27; 0.56)	-0.05 (-0.20; 0.11)
Normal	Deceleration (n= 213)	-0.21 (-0.36; -0.06)	-0.16 (-0.31; -0.02)	-0.15 (-0.29; -0.01)	-0.06 (-0.22; -0.10)	-0.10 (-0.25; 0.06)	0.05 (-0.11; 0.22)
	Normal (n= 533)	Reference	Reference	Reference	Reference	Reference	Reference
	Acceleration (n= 271)	0.52 (0.38; 0.66)	0.31 (0.18; 0.45)	0.44 (0.31; 0.57)	0.14 (-0.01; 0.29)	0.16 (0.02; 0.30)	-0.03 (-0.18; 0.13)
Acceleration	Deceleration (n= 319)	-0.03 (-0.16; 0.10)	-0.07 (-0.20; 0.06)	0.10 (-0.02; 0.22)	-0.02 (-0.17; 0.12)	-0.04 (-0.18; 0.09)	0.17 (0.02; 0.31)
	Normal (n= 316)	0.28 (0.15; 0.42)	0.13 (-0.003; 0.25)	0.30 (0.18; 0.42)	0.13 (-0.02; 0.27)	0.05 (-0.08; 0.19)	0.09 (-0.05; 0.24)
	Acceleration (n= 116)	0.59 (0.40; 0.78)	0.30 (0.12; 0.49)	0.61 (0.43; 0.79)	0.12 (-0.09; 0.33)	0.02 (-0.18; 0.21)	0.11 (-0.10; 0.32)
P value for interaction		.04	.03	.09	.09	<.001	.45

[a]Regression coefficients are linear regression coefficients based on SD scores of the outcome.
[b]Models are adjusted for childhood age at visit and sex. *P* value of interaction term is between fetal and infant growth in model adjusted for childhood age at visit and sex
[c]Regression coefficients are linear regression coefficients based on SD scores of natural log-transformed outcome measures.

© 2019 Vogelezang S et al. *JAMA Network Open.*

eTable 7. Associations of Infant Growth Patterns With Childhood Body Composition

Characteristics	Standard Deviation Scores, Regression Coefficients (95% CI)		
	Body mass index[a,b] (n= 3205)	Fat mass index[b,c] (n= 3185)	Fat free mass index[a,b] (n= 3185)
**Children born small for gestational age**			
PWV, kg/y (n= 161)	0.15 (0.07; 0.24)	0.09 (0.01; 0.18)	0.12 (0.03; 0.20)
BMIAP (n= 151)	0.35 (0.12; 0.58)	0.28 (0.05; 0.50)	0.31 (0.09; 0.52)
AGEAP, mo (n= 151)	0.31 (-0.004; 0.62)	0.26 (-0.05; 0.57)	0.19 (-0.10; 0.48)
**Children born appropriate for gestational age**			
PWV, kg/y (n= 2352)	0.13 (0.11; 0.15)	0.07 (0.05; 0.09)	0.12 (0.10; 0.14)
BMIAP (n= 2207)	0.40 (0.35; 0.45)	0.29 (0.25; 0.34)	0.45 (0.40; 0.50)
AGEAP, mo (n= 2207)	0.07 (-0.01; 0.14)	0.03 (-0.05; 0.10)	0.07 (0.0004; 0.15)
**Children born large for gestational age**			
PWV, kg/y (n= 195)	0.16 (0.10; 0.23)	0.12 (0.06; 0.18)	0.11 (0.05; 0.18)
BMIAP (n= 185)	0.41 (0.26; 0.56)	0.30 (0.14; 0.45)	0.46 (0.31; 0.61)
AGEAP, mo (n= 185)	0.21 (-0.02; 0.45)	0.17 (-0.06; 0.40)	0.13 (-0.11; 0.36)

Abbreviations: PWV, peak weight velocity; BMIAP, body mass index at adiposity peak (calculated as weight in kilograms divided by height in meters squared); AGEAP, age at adiposity peak.

[a]Regression coefficients are linear regression coefficients based on SD scores of the outcome.

[b]Models are adjusted for family-based sociodemographic factors (maternal age and educational level), maternal lifestyle-related factors (pre-pregnancy body mass index, smoking during pregnancy, folic acid use during pregnancy, and parity), and childhood factors (age at visit, sex, ethnicity/race, birth weight, and breastfeeding).

[c]Regression coefficients are linear regression coefficients based on SD scores of natural log-transformed outcome measures.

© 2019 Vogelezang S et al. *JAMA Network Open.*

eTable 8. Associations of Infant Growth Patterns With Childhood Body Composition and Adiposity

Characteristics	Standard Deviation Scores, Regression Coefficients (95% CI)					
	**Body mass index[a,b] (n= 3205)**	**Fat mass index[b,c] (n= 3185)**	**Fat free mass index[a,b] (n= 3185)**	**Visceral fat index[b,c] (n= 2731)**	**Liver fat fraction[b,c] (n= 3058)**	**Pericardial fat index[b,c] (n= 2839)**
**Children born small for gestational age**						
PWV, kg/y (n= 161)	0.16 (0.07; 0.25)	0.10 (0.01; 0.19)	0.12 (0.03; 0.20)	0.10 (0.02; 0.18)	0.12 (0.05; 0.19)	0.03 (-0.04; 0.11)
BMIAP (n= 151)	0.35 (0.11; 0.59)	0.26 (0.02; 0. 50)	0.32 (0.11; 0.53)	0.19 (-0.01; 0.39)	0.31 (0.13; 0.48)	0.05 (-0.14; 0.25)
AGEAP, mo (n= 151)	0.24 (-0.08; 0.57)	0.19 (-0.13; 0.51)	0.17 (-0.12; 0.46)	0.24 (-0.06; 0.552)	0.002 (-0.27; 0.28)	0.08 (-0.21; 0.37)
**Children born appropriate for gestational age**						
PWV, kg/y (n= 2352)	0.16 (0.14; 0.18)	0.10 (0.08; 0.12	0.13 (0.11; 0.15)	0.05 (0.03; 0.08)	0.05 (0.03; 0.07)	0.03 (0.004; 0.05)
BMIAP (n= 2207)	0.47 (0.42; 0.52)	0.34 (0.29; 0.39)	0.49 (0.45; 0.54)	0.20 (0.14; 0.26)	0.09 (0.04; 0.15)	0.16 (0.10; 0.22)
AGEAP, mo (n= 2207)	-0.02 (-0.10; 0.06)	-0.04 (-0.11; 0.04)	0.01 (-0.07; 0.08)	-0.02 (-0.11; 0.07)	-0.02 (-0.11; 0.06)	-0.02 (-0.10; 0.07)
**Children born large for gestational age**						
PWV, kg/y (n= 195)	0.17 (0.10; 0.23)	0.13 (0.07; 0.19)	0.10 (0.04; 0.17)	0.07 (0.01; 0.13)	0.06 (0.01; 0.12)	0.04 (-0.03; 0.10)
BMIAP (n= 185)	0.40 (0.24; 0.56)	0.29 (0.12; 0.45)	0.46 (0.31; 0.61)	0.06 (-0.11; 0.23)	0.09 (-0.07; 0.24)	0.06 (-0.11; 0.23)
AGEAP, mo (n= 185)	0.13 (-0.12; 0.37)	0.09 (-0.15; 0.34)	0.08 (-0.15; 0.31)	-0.10 (-0.35; 0.16)	0.03 (-0.20; 0.27)	0.09 (-0.17; 0.34)

Abbreviations: PWV, peak weight velocity; BMIAP, body mass index at adiposity peak (calculated as weight in kilograms divided by height in meters squared); AGEAP, age at adiposity peak.
[a]Regression coefficients are linear regression coefficients based on SD scores of the outcome.
[b]Models are adjusted for childhood age at visit and sex.
[c]Regression coefficients are linear regression coefficients based on SD scores of natural log-transformed outcome measures.

© 2019 Vogelezang S et al. *JAMA Network Open.*

eFigure. Flowchart of Participants

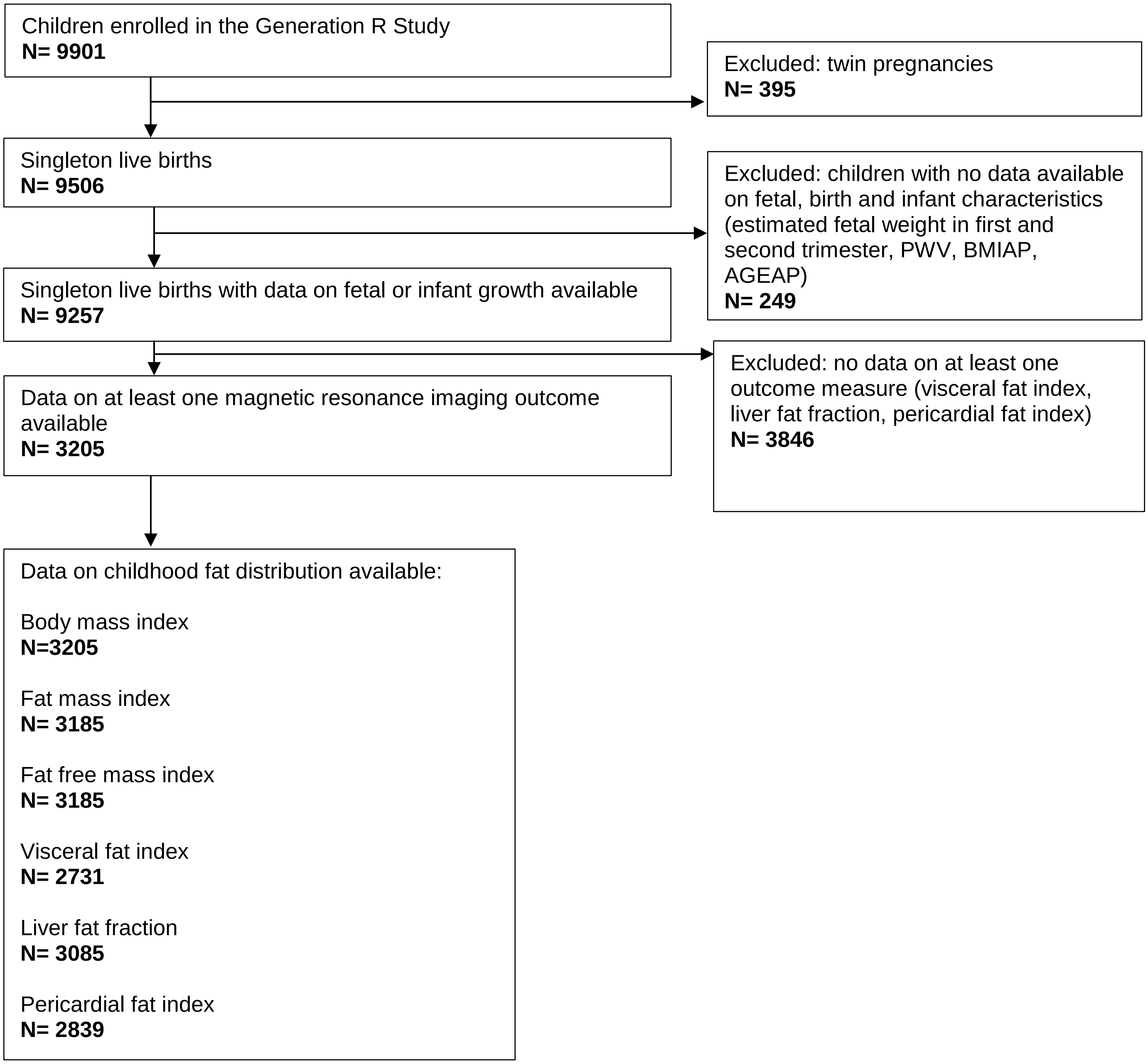

© 2019 Vogelezang S et al. *JAMA Network Open.*
